# Photons Probe Entropic Potential Variation during Molecular Confinement in Nanocavities

**DOI:** 10.3390/e20080545

**Published:** 2018-07-24

**Authors:** Vassilios Gavriil, Margarita Chatzichristidi, Zoe Kollia, Alkiviadis-Constantinos Cefalas, Nikolaos Spyropoulos-Antonakakis, Vadim V. Semashko, Evangelia Sarantopoulou

**Affiliations:** 1 National Hellenic Research Foundation, Theoretical and Physical Chemistry Institute, 48 Vassileos Constantinou Avenue, 11635 Athens, Greece; 2 Department of Chemistry, Laboratory of Industrial Chemistry, University of Athens, Panepistimiopolis Zografou, 15771 Athens, Greece; 3 Institute of Physics, Kazan Federal University, 18 Kremljovskaja str., Kazan 420008, Russia

**Keywords:** entropy, nanothermodynamics, nanoindentation, Atomic Force Microscopy, electric dipole interactions, 157 nm laser, fractal analysis, water contact angle, PHEMA, white light reflectance spectroscopy

## Abstract

In thin polymeric layers, external molecular analytes may well be confined within tiny surface nano/microcavities, or they may be attached to ligand adhesion binding sites via electrical dipole forces. Even though molecular trapping is followed by a variation of the entropic potential, the experimental evidence of entropic energy variation from molecular confinement is scarce because tiny thermodynamic energy density diverseness can be tracked only by sub-nm surface strain. Here, it is shown that water confinement within photon-induced nanocavities in Poly (2-hydroxyethyl methacrylate), (PHEMA) layers could be trailed by an entropic potential variation that competes with a thermodynamic potential from electric dipole attachment of molecular adsorbates in polymeric ligands. The nano/microcavities and the ligands were fabricated on a PHEMA matrix by vacuum ultraviolet laser photons at 157 nm. The entropic energy variation during confinement of water analytes on the photon processed PHEMA layer was monitored via sub-nm surface strain by applying white light reflectance spectroscopy, nanoindentation, contact angle measurements, Atomic Force Microscopy (AFM) imaging, and surface and fractal analysis. The methodology has the potency to identify entropic energy density variations less than 1 pJm^−3^ and to monitor dipole and entropic fields on biosurfaces.

## 1. Introduction

Contrary to classical thermodynamics describing large-scale systems in equilibrium, the mean macroscopic properties of small (nanoscale) systems were unfolded by the introduction of an additional thermodynamic potential, which provides a tool for recounting thermodynamic diversities in small size systems. Today, the perspectives of thermodynamics of small systems [1,2,3,4] are uncovered gradually [5,6], further extended [7,8,9,10], and theoretically [11] and experimentally validated [12]. Diverging responses in micro/nano systems are also coming up from surface localized heterogeneities and topological features, non-extensive fluctuations, boundary, or other constraints, including molecular confinement [13,14,15,16].

Particularly, the restriction of translational, vibration or rotational degrees of molecular freedom within nanoscale regions, either from local confinement of molecules within tiny nanocavities [17], or from electrical dipole interactions between external molecules and ligand adhesive binding sites in a matrix, mainly polymeric, implies major diverging functional responses. Specifically, the outcome of thermodynamic energy flow across two-dimensional (2D) topologies entails the growth of unusual localized surface strain modes at interfaces. Most importantly, in two seminal articles, Gadomski [18] and Gadomski and Rubi [19]—using thermodynamic arguments—pointed out that local 2D topologies, described via the Gaussian curvatures (first, second and mean) of nano/micro entities, are sources of additional Lennard–Jones type (inversely proportional to a distance) and logarithmic type potentials, accordingly, which are associated with local short range electric dipole interactions. This finding is important for biosciences because during irreversible thermodynamic processes, different topological potentials entail diverting thermodynamic/matter responses of the associated fluxes at nanoscale and microscale dimensions.

An intriguing behaviour of 2D surfaces in condensed matter or in life sciences stands up from a nonlinear coupling between electrical and entropic currents in 2D nanoscale semiconductors and uncovers an inherent unidirectional current instability and an existing strong correlation between Euclidian, electrical, and thermal 2D topologies [20]. Also, drug-delivery controversies upon exposure to different molecular size nanoparticles in the extracellular environment are still waiting for a convincing interpretation under the light of thermodynamics of small systems [21].

It is also interesting to note that the stress–strain interface connectivity is directly related to a plethora of morphological shapes that exist in nature. The interplay and flow of different forms of thermodynamic energies across the interfaces of systems [22,23] play a protagonist role, still not widely recognized, in natural evolution, with a sound paradigm that of spontaneous generation of complexity revealed via self-assembled structuring [24]. This universal principle is everywhere in nature—from bimolecular structures [25] to flower-like artificial morphogenetic forms [26] and natural leaves [27].

The stress–strain connectivity from molecular trapping within small size nanocavities is linked to the variation of the entropic potential prior to and after trapping [17,28,29,30,31,32,33]. The entropic potential from the confinement of molecules on a surface might well attain a similar strength as the energy from electrical dipole interactions between the adsorbate external molecular moieties and a ligand binding site in a matrix. In the case of photon processed polymeric matrixes, where both nano/micro cavitations (porosity) and dipole interactions are modified, the key theme is the interplay between an entropic energy variation from the trapping of molecular moieties within nano/micro cavitations of the polymeric matrix, and a diverging electric dipole interaction among the external molecular moieties and the photon modified localized ligand binding site [17].

Indeed, photon-induced processing may alter the physical and chemical properties of organic surfaces and polymers. Particularly irradiation with Vacuum Ultraviolet light (VUV) in the spectral region between 110 to 180 nm implies an extensive modification of physical properties and functionalities of polymeric matrixes via breaking of chemical bonds and emerging of new ones [34,35,36,37,38]. Among others, photon processing of surfaces [39,40] is accompanied by a diversity of parameters, different porosities, sensing efficiencies, chemical stabilities, and excessive surface stressing. Commonly, rather sophisticated experimental approaches and tools are required to obtain the stress–strain surface responses and to identify thermodynamic energy variations in small systems [17,41,42,43,44]. Particularly, the entropic energy variation during sorption of gas analytes within nanocavities formed under laser irradiation of polymeric materials at 157 nm was previously found to be directly correlated with the internal stressing and the strain of the photon processed surfaces [17]. The molecular confinement within the photon-induced nanocavities was revealed experimentally and the strength of an associated entropic nanothermodynamic potential was found to be proportional, among others, to the number of nanocavities.

Thereby, the present work demonstrates the link between the stage of confinement of water vapor molecular analytes within 157 nm laser photon-induced nanocavities in Poly (2-hydroxyethyl methacrylate) (PHEMA) matrixes and an entropic potential levering during sorption. A competition between the entropic potential and a boosted electric dipole thermodynamic potential from the photochemical modification of the matrix is also observed.

The article is organized as follows. First, the efficiency of 157 nm laser radiation on nanocavity formation on PHEMA polymeric surfaces was assessed from the surface analysis (surface parameters) by Atomic Force Microscopy (AFM) imaging. Five different surface fractal analysis algorithms were also used to connect the porosity of the photon processed surfaces with the photon fluence (number of laser pulses) via the fractal dimensionality parameter. AFM Nanoindentation and Water Contact Angle methodologies were also used to measure quantitatively the photon-induced activation of the polymeric surfaces by measuring Young’s modulus, the adhesion force, the contact angle and their time gradients on the photon processed surfaces. Finally, the connection between the thermodynamic entropic and electric dipole potentials (stress) and the surface relative deformation (strain) was identified by White Light Reflection Spectroscopy (WLRS) [17]. The surface stressing was correlated with the level of confinement of the adsorbed water molecular analytes within the photon-induced nanocavities. The methodology, besides chemical sensing, relative strain analysis of materials with sub-nm resolution and thermodynamic energy density flow in the pJm^−3^ range, has the potency to monitor electric dipole and entropic competition in small volumes. Likewise, decrypting the adsorption and diffusive behavior of various molecular analytes on porous materials [45,46,47] either from trapping within tiny cavities, or via shape, surface or volume deformation, might well boost novel potential applications in molecular sensing [37,38,48], gas separation [29,30,49], storage and applications with particular emphasis on nanomedicine [50], bioengineering [51,52], and drug delivery systems [53].

## 2. Materials and Methods

### 2.1. Materials

PHEMA solutions (Mn = 20 K) were prepared from Sigma-Aldrich Co. (St. Louis, MO, USA) 8% *w/w* in ethyl-(S)-lactate. Thin layers (312–387 nm) were also prepared by spin-coating at 3000 rpm for 60 s on Si wafer substrates and finally they were cured at 110 °C at a temperature rate of 0.37 °C s^−1^.

### 2.2. 157 nm Laser

Polymeric layers were placed into a computer-controlled X-Y-Z-θ translation-rotation stage inside a 316 stainless steel chamber and then irradiated by a 157 nm molecular fluorine laser (Coherent Lambda Physik, LPF 200, Santa Clara, CA, USA) under continuous nitrogen flow (99.999%) at room temperature. The laser temporal pulse duration at Full Width at Half Maximum (FWHM) was 15 ns, the repetition rate was 10 Hz, the energy per laser pulse was 28 mJ and the photon fluence per laser pulse was set up at 250 J/m^2^. The ambient pressure in the chamber was kept at 10^5^ Pa. The chamber was purged with flowing nitrogen for 10 min prior to each irradiating step to minimize the presence of oxygen traces. Samples were irradiated at different laser fluence.

### 2.3. AFM Imaging

The AFM imaging and surface analysis were carried out with the diInnova, Scanning Probe Microscope (SPM) Bruker system operating in tapping mode at ambient conditions using a silicon cantilever. The tip radius was 8 nm. High-resolution (512 px × 512 px) and scanning size images 1–5 μm × 1–5 μm were acquired at different scanning areas at the rate of 0.5 Hz. The surfaces parameters of the samples were also evaluated.

### 2.4. Fractal Analysis

The self-affine properties in a certain range of scales are analysed using fractals. Self-affinity is a generalization of self-similarity, which is the basic property of most of the fractals. A part of the self-affine object is similar to the whole object after anisotropic scaling. The fractal dimension (*D_f_*) is a measure of these properties. The fractal dimension, that describes quantitatively the porosity of a surface, was derived from the AFM images by four different algorithms, and the algorithm provided by the AFM “lake pattern” software (diSPMLab Vr.5.01, Veeco, Santa Barbara, CA, USA).

#### 2.4.1. Cube Counting

The cube counting method is derived directly from a definition of box-counting fractal dimension [54,55]. The algorithm is based on the following steps: a cubic lattice with a constant *l* is superimposed on the *z*-expanded surface. Initially *l* is set at *X*/2 (where *X* is the length of the edge of the surface), resulting in a lattice of 2 × 2 × 2 = 8 cubes. Then *N*(*l*) is the number of all cubes that contain at least one pixel of the image. The lattice constant *l* is thus reduced stepwise by a factor of two and the process repeated until *l* equals to the distance between two adjacent pixels. The slope of a plot of log(*N*(*l*)) versus log(1/*l*) gives directly the fractal dimension *D_f_*.

#### 2.4.2. Triangulation

The triangulation method is similar to the cube counting method and is also based directly on the box-counting fractal dimension definition [54]. The method works as follows: a grid of unit dimension *l* is placed on the surface. This defines the location of the vertices of a number of triangles. When, for example, *l* = *X*/4, the surface is covered by 32 triangles of different areas inclined at various angles with respect to the *xy* plane. The areas of all triangles are then calculated and summed to obtain an approximation of the surface area *S*(*l*) corresponding to *l*. Next, the grid size is decreased by a successive factor of 2, as before, and the process continues until *l* corresponds to the distance between two adjacent pixel points. The slope of a plot of log(*S*(*l*)) versus log(1/*l*) gives the number *D_f_* − 2.

#### 2.4.3. Variance Method

The variance method (partitioning) is based on the scale dependence of the variance of fractional Brownian motion [56,57]. In practice, one divides the full surface into equal-sized squared boxes, and the variance (power of Root Mean Square (RMS) value of heights), is calculated for particular box size. The fractal dimension *D_f_* is evaluated from the slope *β* of a least-square regression line fit to the data points in the log–log plot of variance as *D_f_* = 3 − *β*/2.

#### 2.4.4. Power Spectrum

The power spectrum method is based on the power spectrum dependence of fractional Brownian motion [56,57,58]. Every line height profiles forming the image is Fourier transformed, the power spectrum is evaluated and then they are averaged. Fractal dimension *D_f_* is evaluated from the slope *β* of a least-square regression line fit to the data points in the log–log plot of power spectrum as *D_f_* = 7/2 + *β*/2.

The *D_f_* has been calculated for the four different methods using “Gwyddion, SPM data visualization and analysis tool” [59]. Note that the results of the four algorithms differ slightly. This is the result of a systematic error in different fractal analytical approaches.

### 2.5. Nanoindentation

The force-distance (F–D) response of non-exposed/exposed areas was recorded with the di Innova, Bruker system, using phosphorus-(n)-doped silicon cantilever (MPP-11123-10) with a nominal spring constant of 40 N/m at 300 kHz resonance frequency. The Young’s modulus of silicon is typically 127–202 GPa and the Poisson’s ratio is 0.22–0.28. The Young’s modulus of an amorphous polymer in its glassy condition is on the order of few GPa. Therefore, in all the experiments the tip is much stiffer than the sample. By fitting a Hertz model to the force-distance curve, the elastic modulus was calculated using the Scanning Probe Image Processor (SPIP) (Hørsholm, Denmark) force curve analysis software. The hysteresis between approach and retract curves was corrected by the same software. Calculations were performed with Poisson’s ratio 0.5. Data from ten different points on each sample were analyzed.

### 2.6. Water Contact Angle

The chemical modification of irradiated PHEMA surfaces was identified also by the water contact angle of the surfaces under ambient atmospheric conditions. Distilled water droplets of 0.5 μL volume were dropped onto the sample surface from a glass syringe. Water contact angles on samples before and after irradiation and at different time intervals were measured using a contact angle measurement system (Digidrop, GBX Co., Isere, France). The system was equipped with a charged Coupled Device (CCD) camera adequate for static and dynamic contact angle measurements. Droplet images captured at a speed of 50 frames/s. The Digitizer of Droplets, (Digidrop) software was used to fit the profile of captured using the measurements method: tangent at the triple-phase point. Three different contact angle measurements were taken from each sample at different sample positions to calculate the average values.

### 2.7. WLRS

The WLRS measurements were performed by an FR-Basic (ThetaMetrisis™, Athens, Greece) equipped with a Visible-Near-Infrared spectrometer with 2048 pixels and 0.35 nm optical resolution. The light source used in FR-Basic is a white light halogen lamp, with an especially designed stable power supply and soft-start circuit ensuring stable operation over time that is necessary for long time duration experiments. Software controls the tool, performing the data acquisition and film thickness calculations. The samples were spin-coated on native oxide Si wafers having a SiO_2_ layer thickness of 2–3 nm.

## 3. Results

### 3.1. Surface Morphological Characteristics of 157 nm Photon Processed PHEMA Polymeric Matrixes

PHEMA is a polymeric solid material at room temperature, which becomes softer and elastic at higher temperatures. Because PHEMA is a non-toxic material, is used in many biomedical applications including soft contact lenses, implants, controlled drug release, lab on a chip sensor, taste sensors, etc. [51,52,60].

Surface modification of polymeric matrixes by Vacuum Ultraviolet light (110–180 nm-VUV) is a notable method to control surface processing of materials with sub-nm resolution. Early studies on dry PHEMA indicated that it absorbs highly at VUVwavelengths (6.5 μm^−1^). VUV radiation penetrates PHEMA (85%) within an absorption depth less than 1 μm from the surface [36], allowing thus to tailor the surface physicochemical properties and the different functionalities at the micro/nanoscale level, without affecting its bulk properties in a variety of applications. The photon modification of PHEMA hydrogel surfaces, chemistry and topography, including an effective adhering ability of different biocells to attach to the modified areas were previously investigated. Only the outer exposed surface layers of the hydrogel were affected by exposure to 157 nm laser photons, because of water photolysis, including a direct surface water depletion that turns the surface to a hydrophobic one [52].

**Figure 1:**
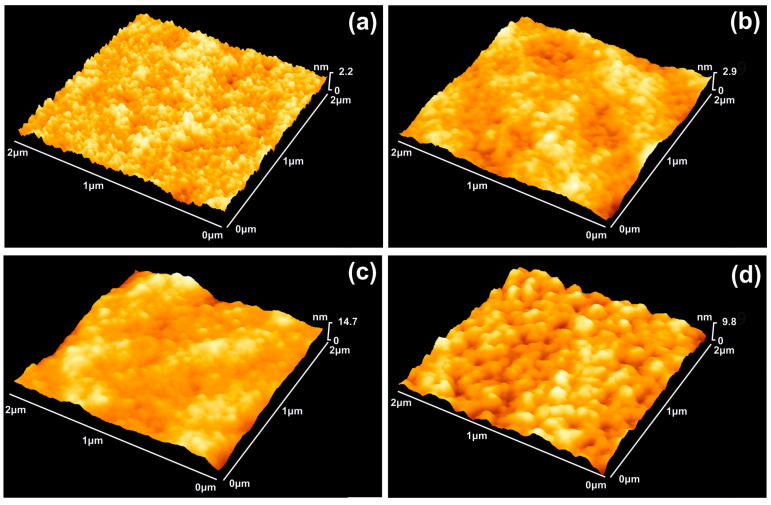
AFM surface imaging of PHEMA layers. The scan area is 2 × 2 μm^2^ and the 157 nm laser fluence 250 Jm^−2^ per pulse: (**a**) Non-irradiated PHEMA layer; (**b**) Irradiation with 20 laser pulses (lp), 5 kJm^−2^; (**c**) 70 lp, 17.5 kJm^−2^; (**d**) 200 lp, 50 kJm^−2^. The structure of the surface is constantly modified under laser irradiation up to 200 lp.

In this work, AFM imaging was used to identify the morphological features of the irradiated PHEMA layers deposited on Si substrates. For the evaluation of the texture characteristics and the cavitation level of the irradiated surfaces, both statistical surface and fractal analytical methodologies were used. The diverging texture characteristics of 2 μm × 2 μm 3D AFM images of non-irradiated and 157 nm irradiated PHEMA surfaces with a different number of laser pulses are shown in Figure 1. Major conformational changes of photon processed PHEMA surfaces are taking place even with surface irradiation with only a few 157 nm laser pulses, also evidenced by a diverging range of surface parameters and sized structures.

The morphology of irradiated areas along the *x*, *y*-axes exhibits different size ~70–140 nm wide islands (compared to ~20 nm islands of the non-irradiated areas) and indicates major photochemical surface changes. The correlation between the surface parameter values and the levering porosity of the surface upon irradiation shows the formation of additional free space in the irradiated matrix. Indeed, because one photon at 157 nm dissociates one chemical bond [35], it is reasonable to accept excesive cavitation and a volume reduction of the matrix.

### 3.2. Surface Analysis

**Figure 2:**
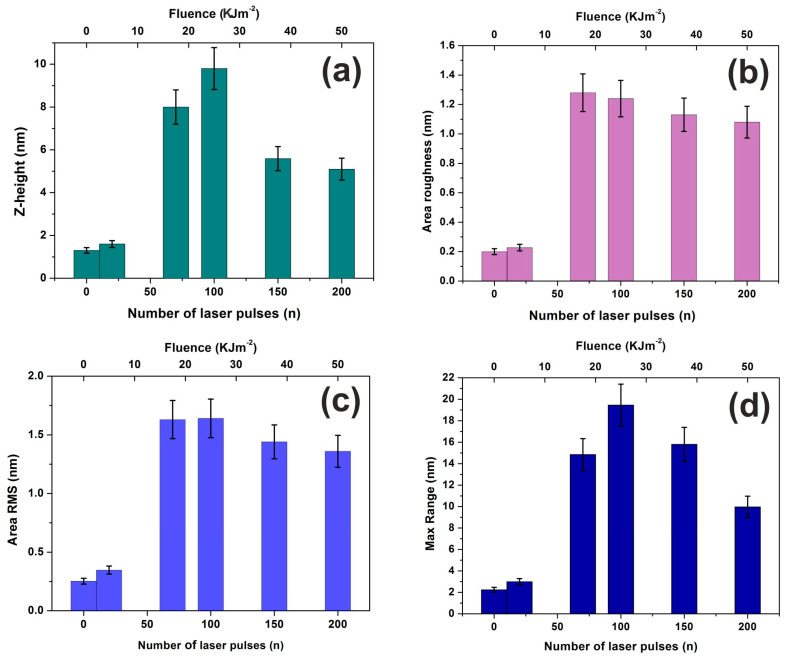
Surface parameters of irradiated PHEMA layers for 2 μm × 2 μm scan areas: (**a**) mean Z-height; (**b**) area roughness (R_a_); (**c**) area RMS; (**d**) maximum range. The surface parameters keep getting larger up to ~100 lp, followed by values reduction up to 200 lp. Then, they remain constant for photon fluence higher than 50 kJm^−2^.

Four different surface parameters were used to quantify the irradiated surfaces and their mean area values, plotted as a function of laser pulses or fluence, Figure 2.

First, the surface roughness histogram or average z-height is an arithmetic mean defined as the sum of all height values divided by the number of data points $\left|Z\right|=\frac{1}{N}\;\sum_{i=1}^{N}Z_{i}$. Next, the R_a_ (area roughness or roughness average) is the arithmetic mean of the height deviation from the image’s mean value, $R_{a}=\frac{1}{n}\sum_{i=1}^{n}\left|Z_{i}-\bar{Z}\right|$. Third the area RMS (R_rms_) is the value defined as the square root of the mean value of the squares of the distance of the points from the image mean value: $R_{rms}=\sqrt{\frac{1}{N}}\sum_{i=1}^{N}{\left(Z-Z_{i}\right)}^{2}$ and finally, the *maximum* range R_max_ is defined as the maximum value of the z-heights. In all examined cases of z-height, Figure 2a, area roughness, Figure 2b, area RMS, Figure 2c and maximum range, Figure 2d, the photon exposed samples were presented with notably increased surface parameter values compared to the non-irradiated samples. The surface parameters values were first rising up to ~100 laser pulses and then were slightly dropped. Because the surface parameters are area size dependent functionals (e.g., the R_rms_ roughness is not an invariant quantity and varies with the size of an image), they are used for a first qualitative evaluation of surface modification upon 157 nm laser irradiation [61].

### 3.3. Fractal Characteristics of 157 nm Photon Processed PHEMA Polymeric Matrixes

**Figure 3:**
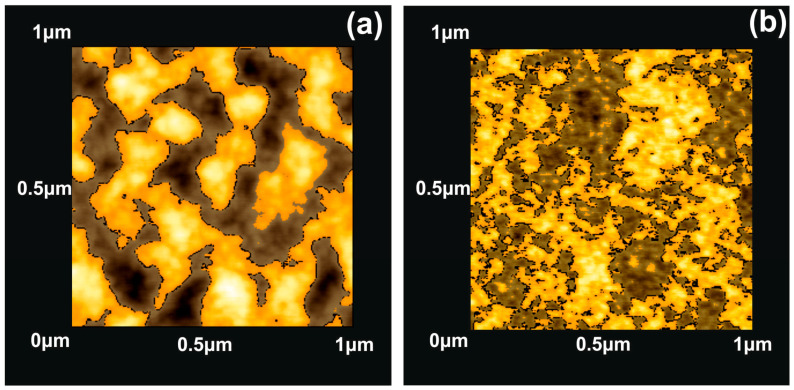
AFM image of ‘‘lake’’ (grey) and ‘‘island’’ (orange) for a fractal area of 2 × 10^3^ nm^2^: (**a**) irradiated with 200 laser pulses; (**b**) non-irradiated.

To bypass the constraints inherent with the surface parameters and for identifying the porous node of surfaces, including a statistical distribution of nanocavities in the polymeric matrix, surface fractal analysis methods were applied. As early works underline, fractal geometry is hidden beneath porous surfaces. The connection between porosity and fractality is based on a statistical “self-similarity” of the empty space topology of the solid conjugates of the matrix after a progressive scaling-down process. Because in porous media the linear, area and volumetric porosities are similar, the need for scaling down the vertical dimension is removed. Therefore, the dimensionality of a surface is equal to two for Euclidean surfaces (nonporous), and equal to three for totally porous surfaces, which retain an entirely fractal character. In the fractal analysis, areas with Z_i_ values above a threshold height Z are known as “islands”, while those with Z_i_ values below the threshold described as “lakes”. At this point is important to note that some AFM software developers denote the “islands” as “lakes”. In this work, the areas with the Z_i_ values higher than the average Z values are named “islands”. A typical “island-lake structure” of nonirradiated/irradiated 1 μm × 1 μm areas with 200 laser pulses is shown in Figure 3.

**Figure 4:**
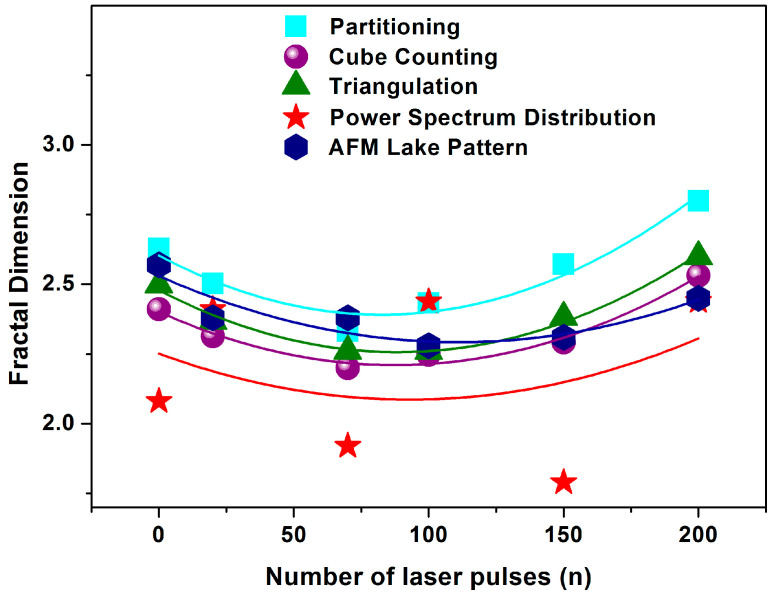
Surface fractal dimension using four different fractal analytical methodologies and fractal shapes (rectangular-partitioning, sphere-cube counting, triangle-triangulation, star-power spectrum) calculated at a different number of laser pulses. The results are compared with those extracted directly by applying the “AFM lake pattern” software provided by the manufacturer. The lines are a guide for the eye.

The mean Z_i_ values were set up at 3.80 and 0.82 nm for the irradiated and non-irradiated layers, respectively, showing diverging surface topologies after 157 nm laser irradiation, making possible quantification of surface porosity and allowing an estimation of surface modification [17,52,53,61]. Two parameters are used to describe the porosity of a surface. One parameter is the “periphery to the area ratio” (PAR) for a set of “islands” or “lakes”, defined as the ratio of the logarithms of the perimeter Π to the area A, where $\Pi =\alpha \left(1+D_{f}\right)A^{\left(1-D_{f}\right)/2}$, and the other is the fractal dimension *D_f_*, associated with both the surface roughness and the topological entropy [18]. To evaluate correctly the porosity of the photon processed PHEMA matrixes, the fractal dimensionality is calculated by applying four different methodologies the partitioning, the cube counting, the triangulation and the power spectrum algorithms [53]. The results are compared with the surface dimensionality extracted directly from the AFM “lake” pattern software, Figure 4.

The 2 μm × 2 μm AFM size images with different laser photon fluence at 157 nm were first digitized to a 512 px × 512 px matrix and then they were processed with the four different fractal analysis algorithms. It is found that the fractal dimensionality is a function of laser photon fluence. All algorithms display a similar trend of fractal dimensionality with the number of laser pulses, even though the fractal dimensionality extracted from the power spectra appears to be slightly different, as expected. It is interesting also to note that the fractal dimensionality, and thus the porosity of the matrix, first declines, attaining its minimum value with 100 laser pulses and then it rises again up to 200 laser pulses, remaining constant afterwards at higher photon fluence, Figure 4.

**Figure 5:**
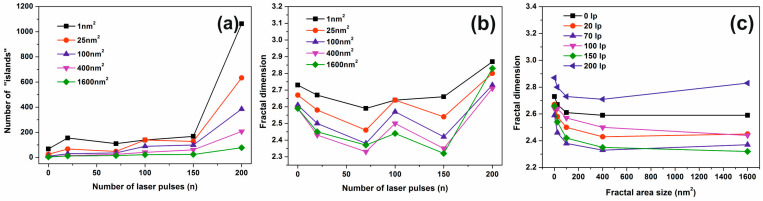
Fractal parameters at different laser fluence: (**a**) number of ‘‘islands’’ for different fractal size vs. number of laser pulses *n*; (**b**) fractal dimensionality vs. number of laser pulses for different fractal size. The concentration of small size nanocavities increases at higher laser fluence; (**c**) fractal dimension vs. fractal size at different number of laser pulses (lp).

Next, the number of “islands” and thus the number of nanocavities with the surface area between 1 and 1.6 × 10^3^ nm^2^ versus the number of laser pulses, Figure 5a is calculated. The number of all the area ‘‘islands’’ remains almost constant between 50–150 laser pulses and then it rises again up to 200 laser pulses, remaining constant afterwards. For a certain number of laser pulses, the generation of small area size “islands” prevails over larger area “islands”, while the concentration of small size “islands” is falling down at higher photon fluence.

**Figure 6:**
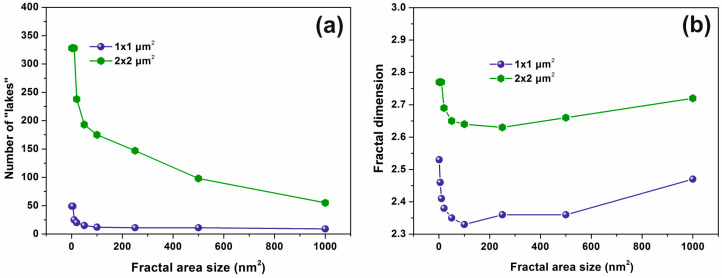
Fractal parameters vs. AFM scanning area of the layer for “lakes”: (**a**) a number of “lakes” vs. fractal area size; (**b**) fractal dimension vs. different fractal area size for two AFM scan areas 1 μm × 1 μm and 2 μm × 2 μm for 200 laser pulses. While the fractal parameters diverging with the scanning area, as expected, the trend of the variation of fractal parameters for different scanning areas remain the same.

The dimensionality of small area “islands” vs. the number of laser photons shows that the small size features contribute a great deal towards a porous like structure, while the porosity between 0–200 laser pulses has a non-monotonous complex structure, Figure 5b. It is again confirmed that independently from the number of irradiating photons, the small size features contribute mainly to the porous character of the polymeric matrix because they possess a relatively higher dimensionality compared to larger ones, Figure 5c. The last statement is again confirmed in Figure 6, where the porous character, of the surface is evident for small size area “lakes”. The number of “lakes” increases for the same “lake” size for larger areas, Figure 6a and the fractal dimensionality rises for larger areas, Figure 6b, indicating a “more porous structure” for larger areas. Similarly, for increasing fractal area size (“lake” size) for both scan areas of 1 μm × 1 μm and 2 μm × 2 μm, the fractal dimensionality decreases up to 100 nm and then it starts to rise again up to 200 nm^2^. Therefore, the number of “lakes” and the fractal dimensionality indicates a size-dependent behaviour of porosity, confirming a porous character for larger size areas, and smaller “lake” sizes up to 100 nm, as expected. Finally, it is worth to notice that the trend of porosity for a given fractal area size falls with the number of laser pulses, Figure 5c, and thus it has the same trend as the relative surface strain (*vide infra*), showing that entropic potential diversity during sorption has its origin in water confinement within different size nanocavities.

### 3.4. Nanoindentation

**Figure 7:**
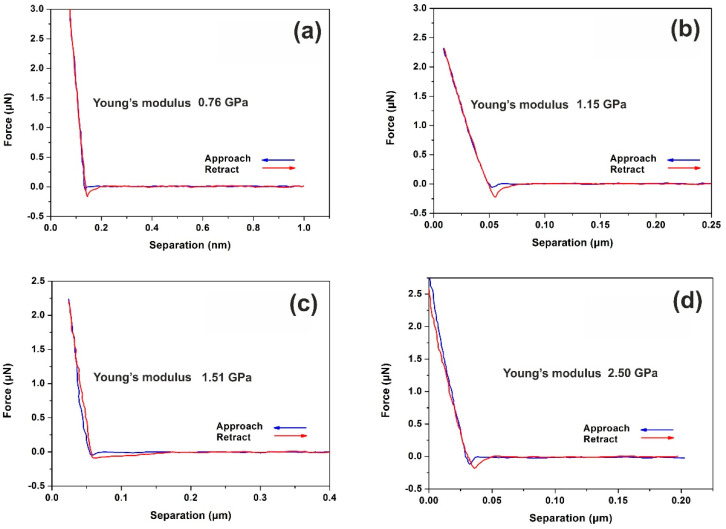
Typical force–distance (F–D) curves of PHEMA thin layer surfaces (312–387 nm) irradiated with a different number of laser pulses (250 J m^−2^ per laser pulse): (**a**) F–D curves of non-irradiated layer; (**b**) F–D curves with 20 laser pulses; (**c**) F–D curves with 100 laser pulses; (**d**) F–D curves with 200 laser pulses.

The mechanical properties of 312–387-nm-thick PHEMA polymers with nanoscale resolution are evaluated by recording the F–D curves [62,63] at different laser photon fluence, Figure 7.

**Figure 8:**
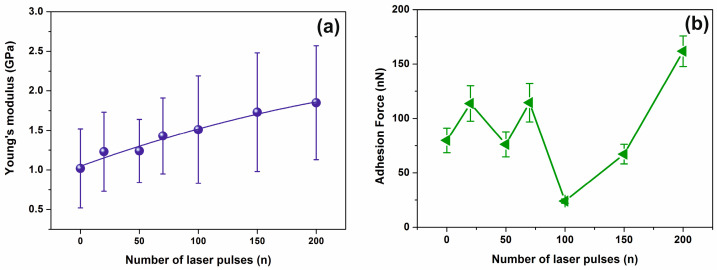
Young’s modulus and adhesion force of PHEMA irradiated layers showing enhanced carbonization at higher laser fluence: (**a**) Young’s modulus; (**b**) adhesion force of PHEMA thin film surface irradiated with a different number of laser pulses (250 J m^−2^ per laser pulse).

Major conformational changes are recorded following the irradiation with only a few laser pulses, evidently indicating large changes of surface adhesion forces, and thus of the surface energy and Young’s modulus of the PHEMA layers, Figure 8a. The differences are attributed to the surface carbonization that always follows photodissociation of polymeric materials.

The reported Young’s moduli in the literature of the non-irradiated PHEMA bulk polymers depend on the hydration conditions. The Young’s moduli of different PHEMA 50–1000 μm thick layers was a function of the hydrogel’s thickness, the cross-linker concentration and the indenter radius and laid between 0.2–1.3 MPa.

Also, Young’s moduli increased notably for film thicknesses smaller than 500 μm [64]. In this work, the elastic moduli of the dehydrated PHEMA of ~1.02 GPa suggest that the layer is shaped in a glassy state [60]. Its average Young moduli increase almost linearly from 1.02 to 1.85 GPa between 0 to 200 laser pulses, Figure 8a. This is the result of both chemical and morphological modification of the surface of the layer by a progressive phase transformation to the carbonized state. In the F–D curves, a downward peak appears in the retraction curve indicating a strong adhesive force between the tip and the PHEMA surface. Additionally, the strength of the adhesive force, measured during the AFM tip retracting phase has a diverging unstable response up to 100 laser pulses, between 25 and 115 nN, and then it rises again at higher laser photon fluence up to 200 laser pulses at ~160 nN, before saturation, Figure 8b, in agreement with the results from surface analysis, Figure 2.

### 3.5. Water Contact Angle

**Figure 9:**
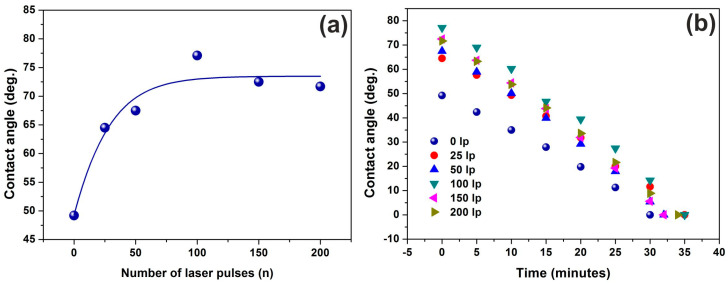
Water contact angle vs. the number of laser pulses showing an increment of surface hydrophobicity at higher laser fluence: (**a**) water contact angle vs. the number of laser pulses; (**b**) time gradient of water contact angle at different time intervals. An identical slope points to a uniform surface response at different fluence, indicating that chemical changes are saturated after a few laser pulses due to the low penetrating depth of the laser radiation at 157 nm.

Water contact angles of PHEMA matrixes were recorded at different photon fluence. They rise from 49° to 71° reaching a saturated plateau at 100 laser pulses and then they remain relatively constant up to 200 laser pulses, Figure 9, with a similar response to Young’s modulus. Also, VUV photon processed PHEMA matrixes attain higher contact angle values at different time intervals, compared with non-irradiated areas, exhibiting hydrophobic surfaces due to carbonization, Figure 9b. The contact angle gradients clearly show the tendency of the PHEMA surfaces to enhance their hydrophobic response by lowering their surface energy [35]. Most important, the non-irradiated/irradiated matrixes show a similar time gradient of contact angles, suggesting similar values of diffusion constants for different porosities.

### 3.6. WLRS

**Figure 10:**
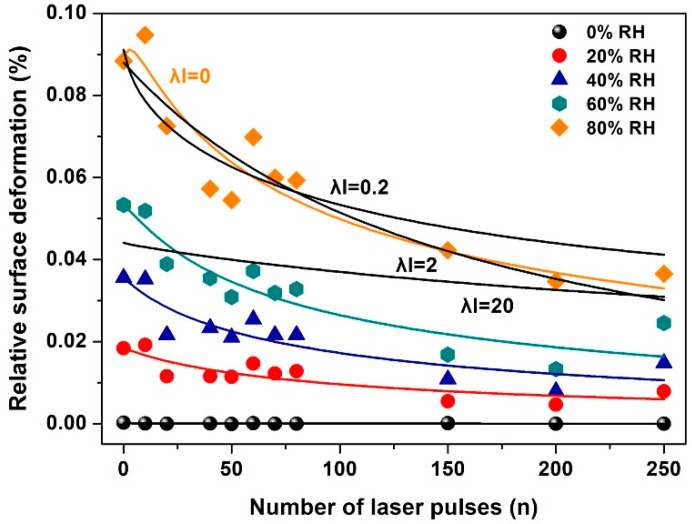
The relative strain of ~345 nm PHEMA thin layers at different irradiating conditions and relative humidity (RH) measured with White Light Reflectance Spectroscopy (WLRS). The black lines at 80% RH are fittings for *λl* values of 0, 0.2, 2 and 20, respectively, suggesting a small contribution (*λl* = 0, 0.2) from electric dipole attachment of water molecules to binding polar sites in the PHEMA matrix.

The relative longitudinal strains of the irradiated PHEMA layers during water vapor sorption were monitored with a sub-nm resolution by WLRS. The relative similar trails of the strain responses of the polymeric matrixes, following irradiation at different laser fluence is increasing with rising concentration of water vapor, Figure 10. For a given value of relative water humidity (RH) the relative strain value decreases with the number of laser pulses. The white light beam registers the relative strain within a cylindrical volume ~*V* = 7 × 10^−14^ m^3^, defined by the cross-sectional size of the white light beam 2.5 × 10^−4^ m and the thickness of the polymeric layer 310–380 nm. The relative surface strain measured with WLRS at 0% and 80% RH of water analytes is 0.0009 and 0.0004, respectively.

## 4. Discussion

The work *δW* made on a thin polymeric layer during an isobaric and isothermal strain $\delta L_{i}$, in the direction of $n_{k}$ outwards from the boundaries of a closed surface *A* in the polymeric matrix is equal with *δW* = $\sigma_{ik}n_{k}\delta L_{i}$. During sorption, the work equals with the free energy variation *δG* of the system prior to and after sorption

(1) $$\delta G=A\sigma_{ik}n_{k}\delta L_{i}=-\delta U-\delta \left(PV\right)+T\delta S+\delta \left(\mu_{i}N_{i}\right)+\delta \left(\Psi Α\right)$$

where *A* is the area surrounding an infinitesimal volume *dV = AdL* and $\sigma_{ik}$ is a second rank stress tensor acting on the volume element *dV*. Its components are related with the force density vector $\vec{f}$ per unit volume element via the equation $f_{i}=\frac{\partial \sigma_{ik}}{\partial x_{k}}$. The term δU on the right side of Equation (1) describes the internal energy of the system, *δ*(*PV*) is the external mechanical work done on the system, which is equal to zero, *δ*(*μ_ι_Ν_i_*) is the chemical energy during sorption, *δ*(*ΤS*) is the entropic energy, and the term $\delta \left(\Psi \left(n\right)\right)=\delta \left[\gamma \left(n\right)+Ε{\left(n\right)}_{s}\iint n_{k}dA_{k}\right]$ is the algebraic sum of the surface energy *δ*(*γ*(*n*)) and the elastic energy strain *E_s_*(*n*) of the nanocavities per unit area, following irradiation with *n* laser pulses. The last term is zero under isothermal and isobaric adsorption conditions, δ(Ψ(n))=0 [65].

For an isobaric and an isothermal change during sorption, the internal energy δU is the electric dipole interaction energy variation.

For a number $N_{b}\left(n\right)$ of photon activated dipole interactive sites within a certain volume *V* in a matrix lesser than the number of analyte molecules $N_{a}\left(n\right)$ adsorbed in the same volume, δU takes the form [66]

(2) $$\delta U=-\lambda lN_{b}\left(n\right)\langle \Phi \rangle =-\lambda lN_{b}\left(n\right)\frac{5d_{xy}^{4}}{64\pi^{4}\varepsilon_{0}\varepsilon_{1}^{2}k_{B}Tr^{6}}$$

where $d_{xy}$ is the *x*, *y* component of the electric dipole moment during the interaction between one analyte molecule and one laser photon activated polymeric site and *r* is their separating distance, *l* is the average number of adsorbed molecules that interact with one photon-activated site of the matrix, which is almost equal to one, and *λ* is the probability that one analyte molecule will overcome a certain energy barrier threshold and eventually will stay attached with an adhesive binding dipole site following a random walk within a period of time shorter than the measuring time duration of strain via WLRS. As the electric dipole moment is of the order of $er_{0}$, where $r_{0}$ is the size of an atomic orbital, $r_{0}=0.01\;\mathrm{nm}$ and the separation distance *r* is of the order of 0.1 nm, then $\langle \Phi \rangle \approx 4.2\times 10^{-21}\;J$.

The difference in the chemical work $\delta \left(\mu_{i}N_{i}\right)$ has a negative and positive contribution from photodissociation of the polymeric site at 157 nm and analytes, because of loss and gain of mass, respectively. Because one VUV photon at 157 nm releases one photodissociated molecule from the matrix, the number of analytes molecules *N_a_* = 4 × 10^12^ within the volume *V* = 7.4 × 10^−14^ m^3^ in the layer (bounded by the cross section of the WLRS beam of ~1.96 × 10^−7^ m^2^, and the thickness of the layer of ~345 nm), for a 50% relative humidity, is comparable with the number of photodissociated moieties ~4 × 10^13^, within the same volume space. Therefore, the relative contribution of the chemical work during sorption in Equation (1) is negligible and hence $\delta \left(\mu_{i}N_{i}\right)\approx 0$. Equally, as each one 157 nm laser photon releases one molecule from the polymeric chain via photodissociation, at the same time one electric dipole-binding site in the remaining molecular polymeric chain is created.

From Figure 1, Figure 2, Figure 3, Figure 4, Figure 5 and Figure 6, it is verified that molecular photodissociation is escorted by a diversity of porosity and nanocavitation. An increasing number of nanocavities $N_{c}\left(n\right)$ is building up in the matrix after each laser pulse and the ratio of the algebraic sum of the number of dipole binding sites and nanocavities to the analyte molecules is

(3) $$x\left(n\right)=\frac{N_{b}\left(n\right)+N_{c}\left(n\right)}{N_{a}}$$

Next, the entropic term is calculated. For $N_{b}\left(n\right)$ polymeric sites and $N_{c}\left(n\right)$ nanovavities, both trapping a number of *N_a_* analyte molecules within a volume defined by the white beam, the number of trapping configurations $\left\{N_{b}\left(n\right)+N_{c}\left(n\right),N_{a}\right\}$ is equal to the number of permutations between the adsorbed analytes and the sum of nanocavities and the dipole adhesive binding sites [17]

(4) $$\begin{array}{c} \left\{N_{b}\left(n\right)+N_{c}\left(n\right),N_{a}\left(n\right)\right\}=\frac{N_{a}!}{(N_{a}-(N_{b}\left(n\right)+N_{c}\left(n\right)))!(N_{b}\left(n\right)+N_{c}\left(n\right))!},\;\mathrm{for}\;N_{b}\left(n\right)+N_{c}\left(n\right)<N_{a}\\ \left\{N_{b}\left(n\right)+N_{c}\left(n\right),N_{a}\left(n\right)\right\}=\frac{(N_{b}\left(n\right)+N_{c}\left(n\right))!}{((N_{b}\left(n\right)+N_{c}\left(n\right))-N_{a})!N_{a}!},\;\mathrm{for}\;N_{b}\left(n\right)+N_{c}\left(n\right)>N_{a} \end{array}$$

Equation (4) was derived using three assumptions. First, one analyte molecule is trapped either from a nanocavity or dipole binding site. Second, the number of adsorbed analytes is fewer than the number of the initially trapped air molecules prior to sorption and third, the isobaric condition implies that the air molecules are replaced by an equal number of analytes. Finally, the entropic term δS after some algebra takes the form

(5) $$\delta S=k_{B}(N_{b}\left(n\right)+N_{c}\left(n\right))[ln\frac{1-x\left(n\right)}{x\left(n\right)}\pm \frac{1}{x\left(n\right)}\ln\left(\left(1-x\left(n\right)\right)m\right)]$$

The positive and negative signs hold for x(n)>1 and x(n)<1, respectively. *m* is the volume ratio occupied by a number of $N_{a}$ analyte molecules in the gas phase outside and within the polymeric matrix and is equal to 1. The relative strain increases from zero at x(n)=1, to its maximum value at x(n)=2, which describes a state of *maximum* entropy variation. For x(n)>2 the relative swelling decreases monotonically and it converges to zero as x(n)→∞. From Equations (1), (2) and (5), the assumptions made and the stress–strain relation $\sigma_{zz}=E\left(n\right)\frac{\delta z}{z}=E\left(n\right)\frac{\delta L}{L}$, the relative surface strain, after some extensive algebra, takes the form

(6) $$\left(\frac{\delta L}{L}\right)={\left(\frac{N_{b}\left(n\right)}{E\left(n\right)V}\right)}^{\frac{1}{2}}\;[-\lambda l\langle \Phi \rangle +K_{B}T\left(1+\beta \left(n\right)\right)[ln\frac{x\left(n\right)}{1-x\left(n\right)}\pm \frac{1}{x\left(n\right)}\ln\left(1-x\left(n\right)\right)]{]}^{\frac{1}{2}}$$

where *E*(*n*) is the Young modulus of the surface and $\beta \left(n\right)=\frac{N_{c}\left(n\right)}{N_{b}\left(n\right)}$.

Equation (6) is the final result. It relates the experimentally measured relative strain of the surface $\left(\frac{\delta L}{L}\right)$ and Young modulus E(n) with the number of nanocavities, the photon induced dipole adhesive binding sites in the matrix and the water vapor molecules via x(n).

The first term on the right in Equation (6), −λl 〈Φ〉, is the contribution of the electric dipole interaction between the dipole adhesive binding sites and the adsorbed molecules in the stressing field and the third and fourth terms describe the stressing field from the entropic variation during the sorption of analytes and their confinement within nanocavities.

To fit Equation (6) to the experimental data of Figure 10, the functional dependence of *x*(*n*) on the number of photons *n* has to be determined. Because *x*(*n*) is proportional to the number of dipole binding sites and the number of nanocavities following photodissociation of the polymeric matrix, *x*(*n*) is a measure of the surface carbonization via the term *N_c_*(*n*). Therefore, *x*(*n*) and the Young modulus *E*(*n*) carry the same functional dependence with the number of 157 nm laser pulses, in agreement with previous results for PDMS polymers [17]. By using a linear functional for both *x*(*n*) and *E*(*n*), the best fit of Equation (6) to the experimental data of Figure 10 is for β(n)=0.2 and 0≤λl<0.1, indicating a small contribution in the surface strain from electric dipole interactions. 

## 5. Conclusions

The restriction of translational, vibration, or rotational degrees of molecular freedom within tiny space regions, either from the local confinement of molecules within tiny nanocavities or from electrical dipole binding between external molecules and ligand adhesive binding sites implies major diverging functional responses from bulk counterparts. An entropic jump trails the confinement of water vapor molecules adsorbates in either nanocavities or in electric polar binding sites crafted by 157 nm laser light on PHEMA surfaces. The entropic variation is probed by white light reflectance spectroscopy that monitors the relative strain of the polymeric surface (swelling/deswelling) by displaying the surface relative strain prior to and after sorption of water molecules. The sub-nm surface strained field is a function of the number of nanocavities, the water molecules and the Young modulus of the surface. The methodology permits the development of nanothermodynamic sensors that monitor polar-entropic completion and nanothermodynamic energy flow below pJm^−3^ energy density range for bioapplications.
